# High Output Heart Failure Secondary to Aorto-Caval Fistula Treated With an Amplatzer Septal Occluder: Case Report and Review of Literature

**DOI:** 10.7759/cureus.14430

**Published:** 2021-04-12

**Authors:** Fida Charif, Pierre Nassar, Dani Youssef, Ziad Neghawi, Mohamad Saab

**Affiliations:** 1 Pulmonary Critical Care Medicine, Beirut Cardiac Institute, Beirut, LBN; 2 Adult Cardiology Division, Beirut Cardiac Institute, Beirut, LBN; 3 Pediatric Cardiology, Beirut Cardiac Institute, Beirut , LBN; 4 Radiology Division, Beirut Cardiac Institute, Beirut, LBN; 5 Cardio-vascular Surgery Division, Beirut Cardiac Institute, Beirut, LBN

**Keywords:** aorto-caval fistula, amplatzer septal occluder, high output heart failure

## Abstract

Aorto-caval fistula (ACF) is a rare cause of high output heart failure (HOHF). 80 % of cases are due to ruptured abdominal aortic aneurysm, while 10 to 20% are traumatic or congenital. Early diagnosis and treatment are crucial in order to prevent the progression to HOHF. Open surgical repair has been the mainstay therapy of arterio-venous fistulas including aorto-caval fistula; however endovascular approach has become an evolving therapeutic option in the last 20 years. Here, we present a case of high output heart failure secondary to traumatic aorto-caval fistula due to shrapnel injury to the abdomen. Our patient was managed with endovascular approach by the deployment of amplatzer septal occluder that excluded completely the fistula, resulting in the progressive improvement of HOHF. In this manuscript, we review etiologies of high output heart failure and summarize cases of aorto-caval fistula treated with amplatzer septal occluder reported in literature. We also highlight the importance of this endovascular device in the presence of metallic foreign body in the aorta.

## Introduction

High output heart failure (HOHF), unlike other forms of heart failure (reduced or preserved ejection fraction), is defined as cardiac output (CO) greater than 8L/min or cardiac index (CI) greater than 4.0 L/min/m^2^ along with a low systemic vascular resistance [1]. HOHF is due to increase oxygen demand as a result of hypermetabolic state (e.g exercise, hyperthyroidism, fever, sepsis, pheochromocytoma); or increase cardiac blood flow due to a bypass of arteriolo-capillary bed like arterio-venous fistula (AVF) including aorto-caval fistula (ACF). The ensuing increase in cardiac preload may dramatically increase the cardiac output, mechanical shear stress, remodeling, and cardiac dilatation leading to progressive right and left ventricular failure [2, 3]. Open surgical repair has been the mainstay therapy of AVF, however, the endovascular approach, which was first described in 1999 [4], has become an evolving therapeutic option in the last 20 years with lower mortality and morbidity rate compared to the conventional surgical approach [5]. Amplatzer septal occluder (ASO) is a well-known endovascular treatment approach for intra-cardiac shunt [6], however, its use for the treatment of ACF has been under-reported and was firstly described by Francois Godard et al in 2005 [7]. Here, we report the case of traumatic ACF leading to HOHF and managed successfully by ASO. 

## Case presentation

**Figure 1:**
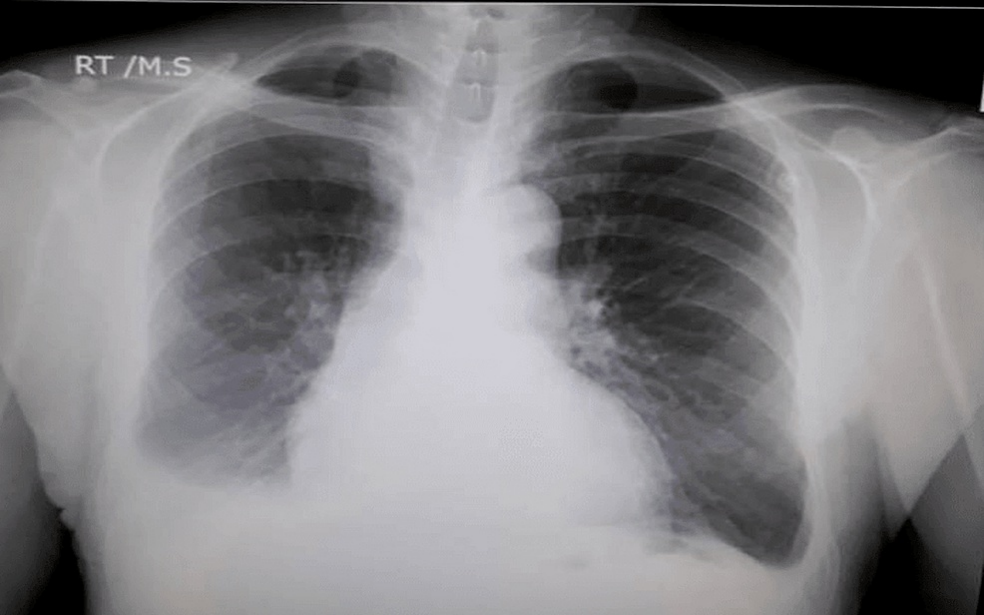
Chest radiograph PA view Shows mild right pleural effusion, mild cardiomegaly and dilated right heart cavities

A 24-year-old man presented to our hospital with four months history of progressively increasing signs and symptoms of right-side heart failure. He had an abdominal shrapnel injury to the abdomen for which he underwent a partial colectomy via a laparotomy five months prior to the beginning of the first symptoms of shortness of breath. On admission his physical examination was remarkable for tachypnea (24/min), pulse oxygen saturation 92% on room air, neck vein distension, markedly decreased breath sounds over the right lung field. The abdominal examination showed a periumbilical palpable thrill with a systolo-diastolic murmur. We also noted bilateral lower limb pitting edema. His chest x-ray showed enlarged right heart cavities and right pleural effusion [Figure 1].

**Figure 2:**
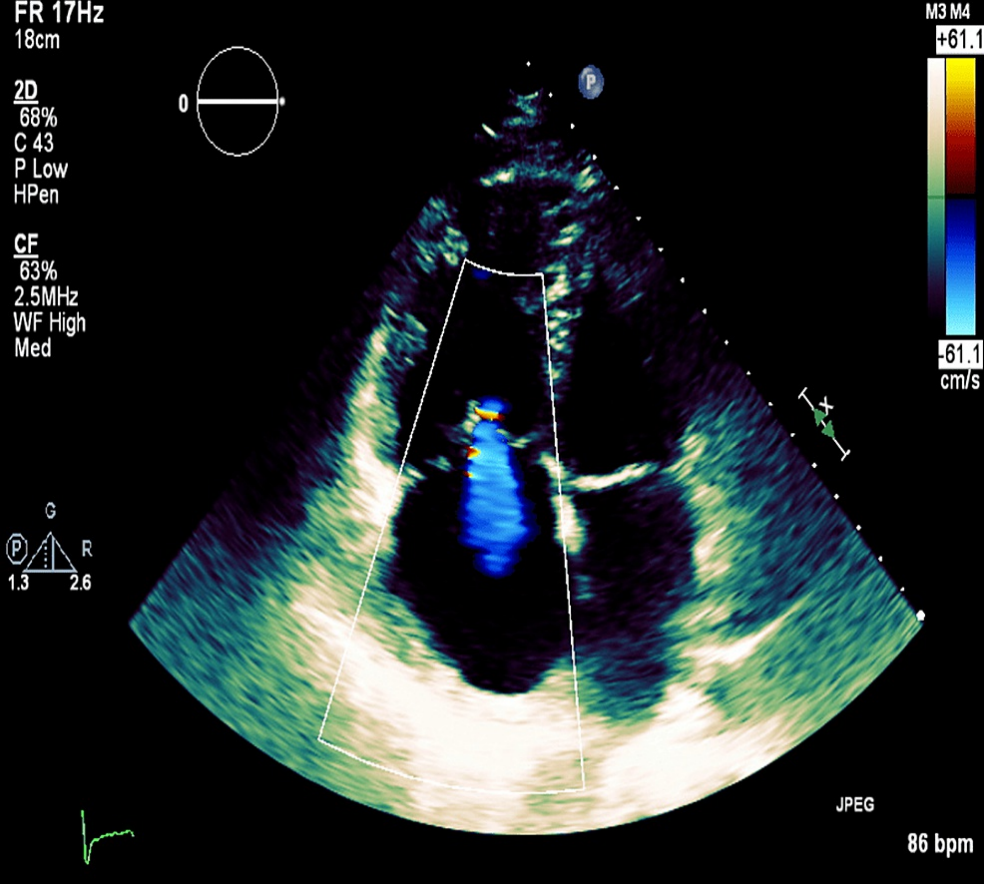
Transthoracic echocardiogram Transthoracic echocardiogram Four chamber view shows severe dilatation of right heart cavities.

The echocardiography showed severely dilated right heart cavities with mildly impaired systolic function (TAPSE 14 mm) [Figure 2]. 

**Figure 3:**
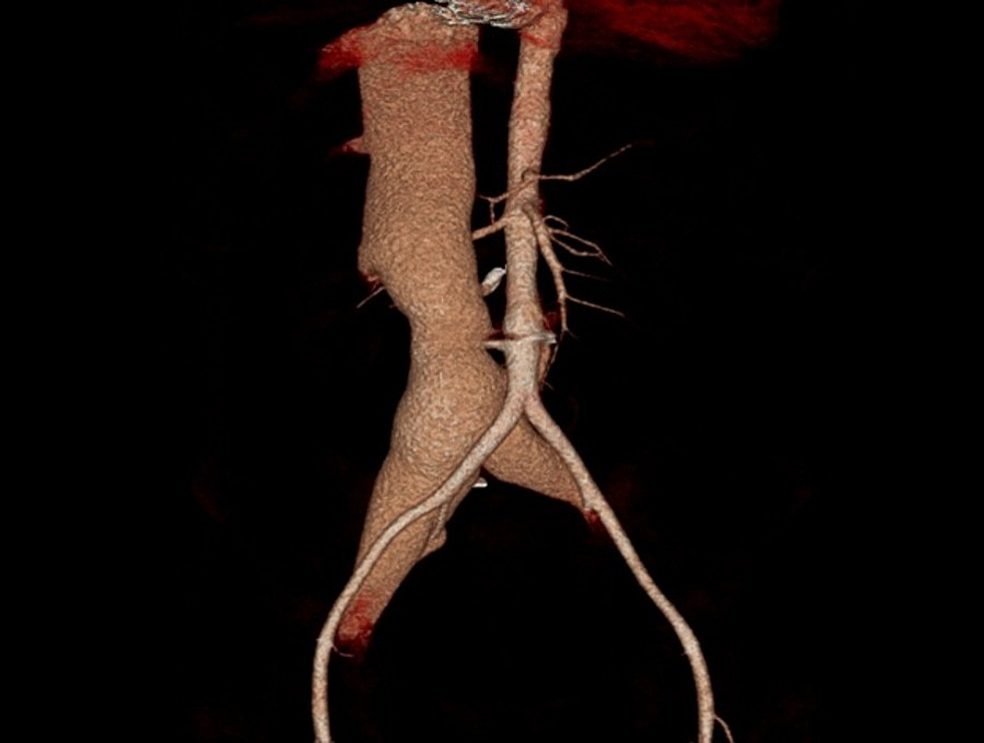
Computed tomography angiography (volume rendering) A communication between the aorta ( just above the aortic bifurcation) and the inferior vena cava. It shows the severely dilated inferior vena cava.

**Figure 4:**
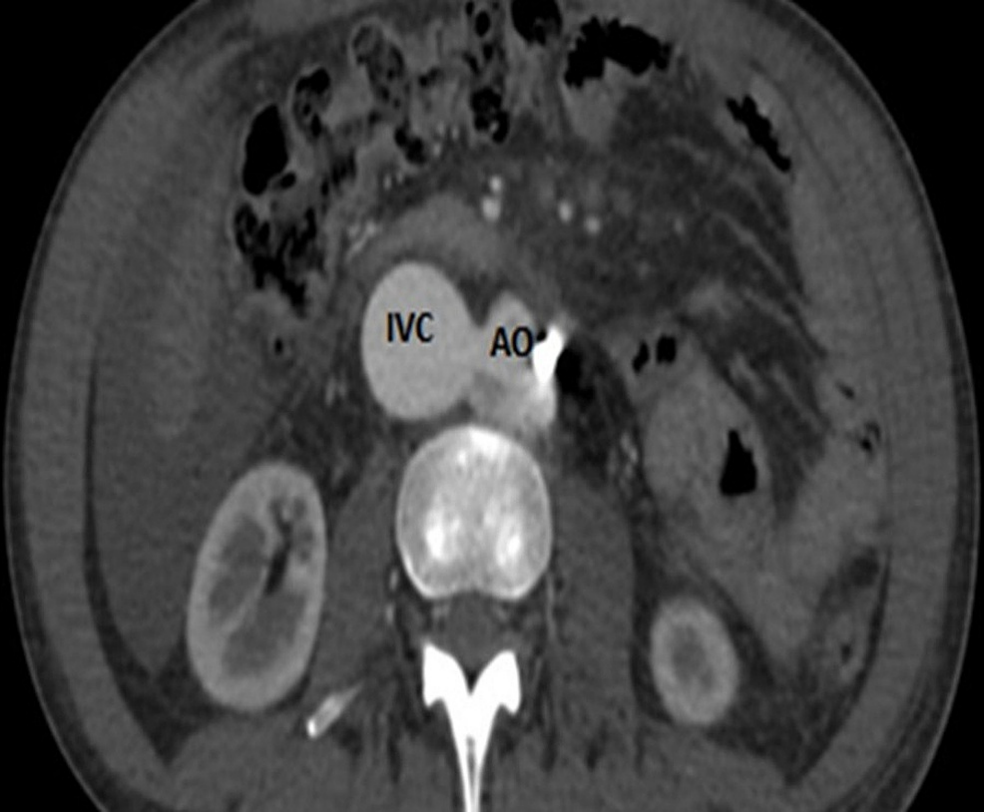
Computed tomography angiography (Axial view) Shows the communication between the aorta (AO) and the inferior vena cava (IVC) during the arterial phase. we also note the presence of the foreign material in the AO.

We also noted mild tricuspid valve regurgitation. Left side chambers and valves were normal. A right heart catheterization (RHC) showed a high cardiac output, cardiac index and markedly decreased systemic vascular resistance and severe pulmonary hypertension [central venous pressure (CVP) 18 mmHg, CO 10.8 L/min, CI 6.07 L/min/m^2^, systemic vascular resistance (SVR) 348 Dynes/sec/cm^-5^, mean pulmonary artery pressure (mPAP) 45mmhg, pulmonary vascular resistance (PVR) 460 Dynes/sec/cm^-5^]. A computed tomography angiography of the abdomen (CTA) showed an ACF of (1.2 cm) of a diameter just above the aortic bifurcation with severe dilatation of the inferior vena cava (46 mm) and the right iliac vein, we also noted the presence of remaining shrapnel in the aorta [Figure 3, 4]. 

All these findings were consistent with the diagnosis of HOHF secondary to ACF due to a remote abdominal shrapnel injury. After a multidisciplinary discussion of our cardiovascular team, we decided to close the ACF with an ASO device. After a double puncture of the left femoral artery and the right femoral vein, a pigtail catheter was introduced through the left femoral artery and positioned in the aorta just above the ACF. While the angiography visualized the large ACF, the delivery system was introduced through the left femoral vein reaching the aorta via the ACF, and then the ASO device (occlutech 10.5 mm) was deployed, releasing the left disc in the aorta and the right disc in the inferior vena cava [Video 1]. 

**Video 1:** Deployment of the Amplatzer Septal Occluder Angiography shows the deployment of the amplatzer septal occluder

**Figure 5:**
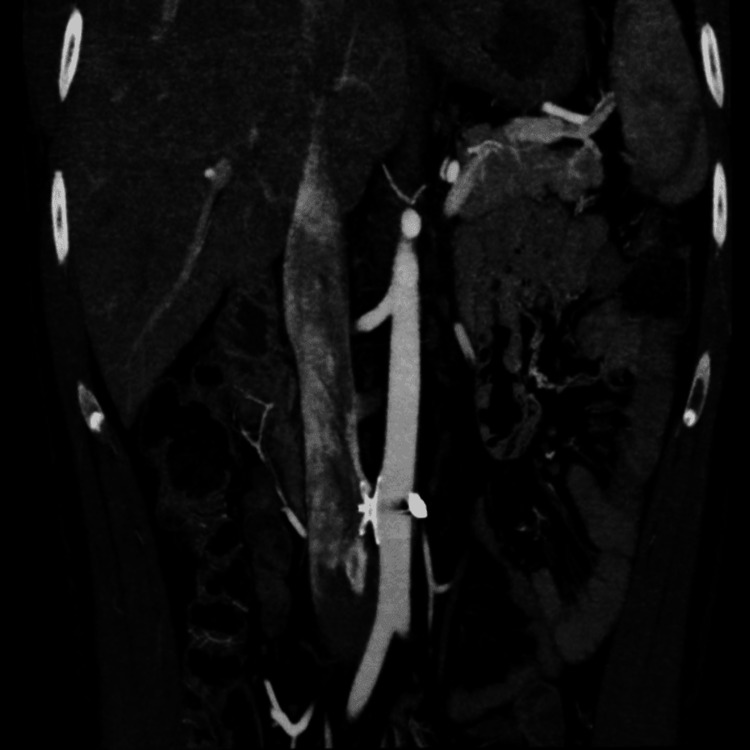
Computed tomography angiography of the abdomen (coronal view) Exclusion of the fistula with mild endoleak and well positioned ASO.

**Figure 6:**
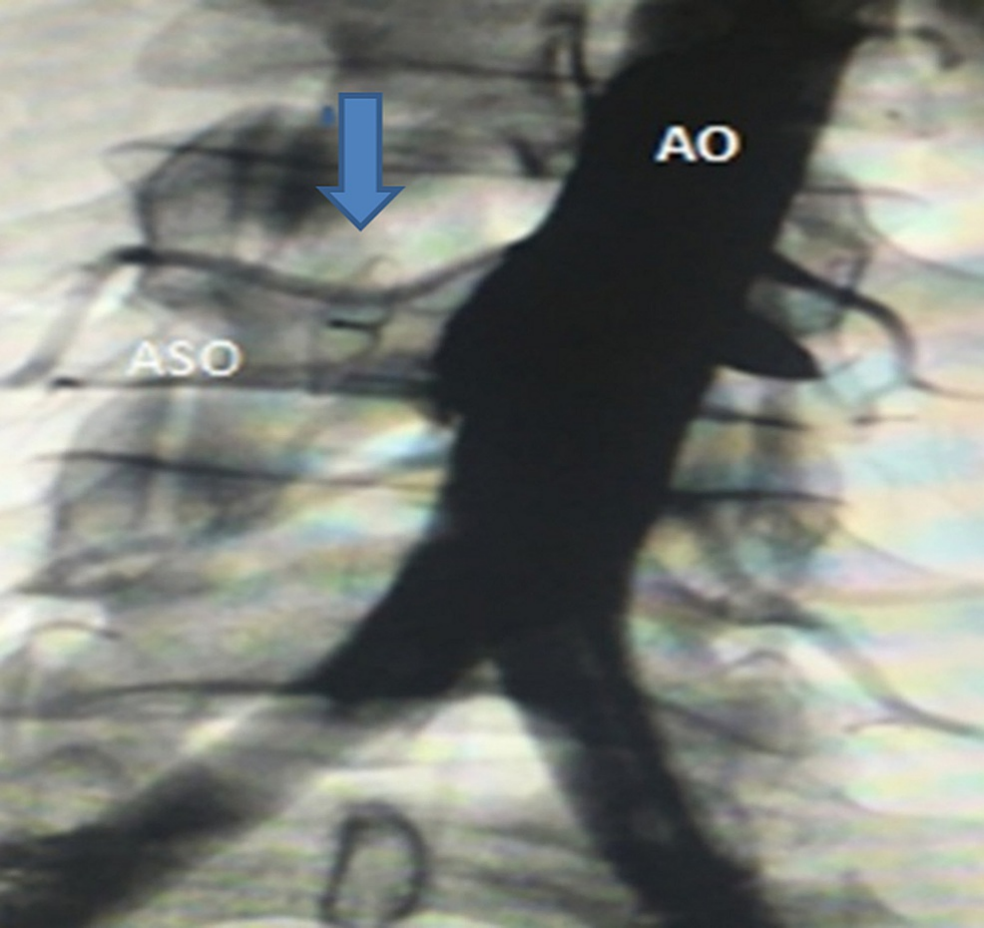
Angiography of the aorta Complete exclusion of the Aorto-caval fistula with stable position of the amplatzer septal occluder at 6 months follow up

An angiography showed the well-positioned ASO device and a mild persistent endoleak. A few days later, our patient’s symptoms markedly improved, and a normal abdominal examination with complete disappearance of the thrill and the systole-diastolic murmur. Repeat echocardiogram showed markedly decreased diameter of right heart cavities, inferior vena cava (26 mm), improvement of right ventricular systolic function (TAPSE 18 mmHg), and disappearance of tricuspid regurgitation. CTA at three months and angiography at six months follow-up showed complete exclusion of the ACF [Figure 5] and well-positioned [Figure 6]. Repeat RHC at six months confirmed the resolution of HOHF (CVP 4mmhg, CO 4.2 L/min, CI 3L/min/m^2^, SVR 1200 Dynes/sec/ cm^-5^, mPAP 18 mmHg, PVR 150 Dynes/sec/cm^-5^ )

## Discussion

ACF is one of the very rare causes of HOHF. 80-90% of ACF are due to ruptured abdominal aortic aneurysm (AAA) into the inferior vena cava, although the incidence is only 2-7% of all cases of AAA; while traumatic and iatrogenic causes represent 10-20% [8]. Clinical manifestations include back pain, symptoms, and signs of HOHF, abdominal bruit, palpable abdominal mass, peripheral edema, liver, and renal failure. Traumatic ACF was first described in 1831, while Cooley et al [9] reported the first successful surgical repair in 1954. Careful history and physical examinations raise the suspicion of ACF. Diagnosis is usually confirmed by duplex ultrasound and or computed tomographic angiography (CTA). Dabbouseh NM et al [10], Srisuwan T et al [11], Waldrop JL et al [12], reported three cases of ACF secondary to penetrating trauma. The authors described a delayed diagnosis and successful endovascular repair of ACF. Recent reports showed lower mortality and morbidity rate compared to the conventional surgical approach [5]. However, a contemporary review of both endovascular and open ACF repair showed that the endovascular approach was not associated with lower complications or mortality rate [13]. These results were attributed to the higher rate of endoleak (50%) and delayed ACF diagnosis reported by the cases included in this review. The use of ASO to treat a residual ACF following stent deployment in patients with ruptured AAA has also been reported [14]. Our patient was treated by the deployment of ASO; we estimated that the deployment of a stent-graft would be at risk of rupture by the remained shrapnel in the aorta post abdominal injury. The other option was the vascular plug which was excluded due to its small size, the disadvantage of being compressed and protruding into the aorta or IVC. The use of ASO to treat ACF was first reported by Francois Godard et al [7] who described the first two cases of ACF that were treated successfully with ASO. Furthermore, ACF complicating a stent-graft deployment during an AAA repair has been also described and was therefore successfully treated with ASO [15, 16]. ASO is an effective therapeutic option of ACF, especially in patients post-traumatic abdominal injury with remaining shrapnel material.

## Conclusions

HOHF and ACF are both rare and reversible conditions. ASO seems to be a promising endovascular technique for the treatment of ACF and/or exclusion of a residual endoleak following a stent-graft deployment. Our case highlights the importance of this endovascular device in the treatment of ACF especially In the presence of metallic foreign body (shrapnel) in the aorta, since the use of a stent-graft may lead to its fracture during deployment. Further studies using the ASO for treatment of ACF are needed. 
